# Modeling egg production as a means of optimizing dietary nutrient contents for laying hens

**DOI:** 10.1093/af/vfz010

**Published:** 2019-03-30

**Authors:** Nilva Kazue Sakomura, Matheus De Paula Reis, Nayara Tavares Ferreira, Robert M Gous

**Affiliations:** 1 Faculdade de Ciências Agrárias e Veterinárias, Unesp Univ Estadual Paulista, Jaboticabal, Sao Paulo, Brazil; 2 Department of Animal and Poultry Science, University of KwaZulu-Natal, Pietermaritzburg, South Africa

**Keywords:** feed intake, modeling, optimization, poultry nutrition, simulation

**Implications**

- Simulated models or descriptions of how laying hens or broiler breeders respond to dietary contents, while taking account marginal costs and revenues, are invaluable in determining how to maximize or minimize the margin over feed cost, feed conversion efficiency, number of eggs produced, or chicks hatched per hen for any given commercial operation.
- For commercial laying hens and broiler breeders, the theory is applied to each individual making up the population using appropriate statistics for specific parameters to produce a stochastic model.
- A simulation modeling approach can eliminate the need for expensive, long-term laying trials designed to measure the response of laying hens or broiler breeders to energy and protein.

## Introduction

Poultry geneticists have been highly successful in improving the egg output and feed efficiency of laying hens and, to a lesser extent, broiler breeders. As the potential output of these birds has changed so have their nutrient requirements and these are reflected in user manual guidelines, tables of requirements, and company know-how. However, the basis of these suggested changes is unlikely to be based on theoretical calculations of the updated requirements of the bird due to the lack of a robust theory describing the manner in which the nutrient requirements are calculated. Such a theory has been developed recently and is described in this article.

To calculate the amino acid and energy requirements of an individual laying hen, it is necessary to be able to predict its potential body protein weight at the start of lay and its potential egg output and egg weight over time (Emmans and Fisher, 1986). From this information, and the nutritional constants that describe the rates of conversion of feed nutrients to maintenance and egg nutrients, it is possible to calculate the daily requirement of each of the amino acids and energy throughout the laying period. Once the requirements for an individual hen are correctly calculated, the same process can be followed for each bird in a simulated flock, the parameters for each bird being defined using the means and standard deviations of each parameter describing the mean individual.

Calculating the daily nutrient intake required to meet the potential performance of each individual in terms of numbers of eggs produced over the production cycle would improve the possibility of optimizing the composition (and daily allocation, in the case of broiler breeders) of the feed offered to the flock. To achieve these goals, a comprehensive understanding of the factors influencing the attainment of sexual maturity in these birds, of the ovulatory cycle and how this changes during the laying cycle, and of the changes that occur in egg and body component weights over time is required.

This article describes such a stochastic egg production model developed at Universidade Estadual Paulista (UNESP), Brazil. In this article, only the calculation of the nutrient requirements of flocks of laying hens and broiler breeders is described, with examples of these requirements for simulated populations having different potential outputs. No attempt has been made to use this information to predict food intake or the consequences on performance of situations where hens were unable to consume sufficient feed.

**Figure 1.**
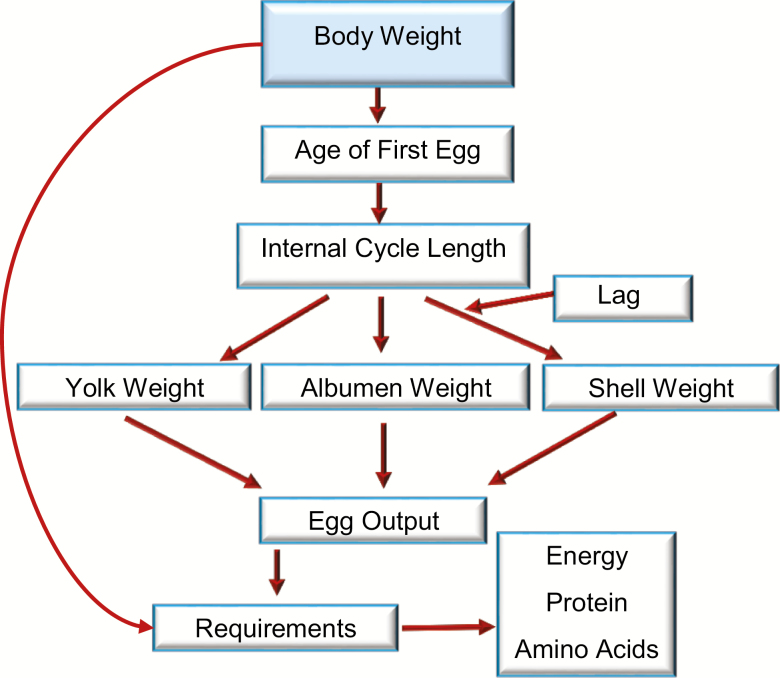
Flow diagram from the dynamic simulation model to estimate the nutritional requirements as a function of body protein weight and egg production.

The model was developed based on studies conducted at the Poultry Science Laboratory, UNESP, Brazil. One study was conducted with two strains of laying hens (Hy-Line W-36 and Isa brown) (Bendezu et al., 2018) and another with broiler breeders (Cobb) (Ferreira et al., 2016) to generate the equations and parameters used in the models described herein. An overall scheme of the egg production model is presented in Figure 1.

## Predicting Performance

The age and body weight of a bird on the day it lays its first egg have a very strong influence on future egg weight and the number of eggs laid, which are important considerations for both the layer and broiler breeding industries. These characteristics can be modified by lighting and/or the nutritional control of growth. In full-fed, egg-type hens, a 10-d delay in sexual maturity that has been achieved through a lighting program results in an increase of 1.3 g in mean egg weight and a reduction of seven eggs over 52-wk lay, but the total egg output will be similar (Lewis and Morris, 2006). The age at sexual maturity of a hen must be calculated to predict the laying performance of a hen.

Important considerations in predicting sexual maturity in hens are that gonadal development takes place in whatever lighting program is used, that lighting modifies the age at sexual maturity, that changing photoperiods has a greater influence than does constant photoperiod (Lewis and Morris, 2006), and that the response of a broiler breeder to light differs from that of a commercial laying hen because broiler breeders, unlike commercial laying hens, exhibit photorefractoriness (Lewis et al., 2003). The attainment of sexual maturity is therefore under both genetic and environmental influences, with broiler breeders still exhibiting photorefractoriness, while this has been eliminated by genetic selection in laying pullets (Lewis et al., 2007).

In full-fed commercial pullets, lighting is the most important environmental factor influencing age at first egg (Lewis et al., 2002). When pullets are reared under constant daylengths, the length of the photoperiod used can influence age at first egg (Lewis et al., 1998), and when one or two changes are made to the daylength during rearing, the length of each photoperiod also has an influence (Lewis and Perry, 1994; Lewis et al., 1996). Although the initial and final photoperiods are the principal components of a lighting program influencing age at first egg in full-fed pullets, the effects of a given change in photoperiod are not the same at all ages (Lewis and Morris, 2006). Also, the advance in age at first egg for birds started on 8-h photoperiods and, given a single increment in photoperiod at a defined age, is proportional to the size of the increment up to about 13 h (Lewis et al., 1998), but not for longer final photoperiods (Lewis et al., 1996).

Lewis et al. (2002) proposed a model to predict age at first egg of full-fed pullets when changes were made to the photoperiod during rearing. The four components of the empirical model, each of which is calculated separately, deal with 1) the genetic differences in age at first egg in birds maintained on constant photoperiods from hatching; 2) the change in age at first egg as a function of age at transfer to the final photoperiod; 3) the acquisition of sensitivity to increases in photoperiod in the young pullet; and 4) the onset of spontaneous rapid gonadal development, that is, the proportion of birds maturing under the influence of the initial photoperiod, without responding to a late change in photoperiod.

Broiler breeders were found to respond differently to light than commercial laying hens (Lewis et al., 2003). The condition of photorefractoriness prevents broiler breeders from being photoresponsive until at least 10 wk of age, and to cause some individuals still to be photoperiodically nonresponsive at 18 wk, so their age at maturity must be calculated differently from the method used for laying pullets. Their age at first egg is predicted using the theory proposed by Lewis et al. (2007), where three equations are used to estimate the age of sexual maturity in a flock of birds (average age when 50% of the flock are laying), taking account of body weight at 20 wk of age and the light program used during the rearing phase.

Describing the potential rate of lay of a laying hen is complex because of the number of interacting factors involved, and the fact that the potential varies over time within each individual. The mathematical model of Etches and Schoch (1984), based on the theory of Fraps (1955), demonstrated that two functions, representing two independent but interacting systems of the hen’s asynchronous ovulatory cycle, were able to predict realistic ovulation times and intrasequence ovulation intervals. Johnston and Gous (2003) extended this model by defining a set of continuous functions, representing the changes required to the values of the different parameters, such that the prediction of any sequence length is possible.

Mean rate of lay in a flock of hens at a particular age is determined by the individual patterns of sequential laying at that time. Within a population of birds, individuals of the same age show considerable variation about a mean sequence length, which may be due to variation in the length of the open period for luteinizing hormone release or variation in follicular dynamics (Lewis and Morris, 2006). This variation may be accounted for using mean values and standard errors for each of the parameters in the model (Johnston and Gous, 2003). Such a population of birds would generate a range of ovulation times, the distribution of which is unimodal and positively skewed in young hens, becoming bimodal with age. Reproductive senescence in hens manifests as an increase in the intrasequence ovulation and oviposition intervals with time, as well as an increase in the number of pause days.

Different approaches have been used to model the decline in rate of lay over time. Essentially, there is evidence to show that sequence length tends to rise initially (Lewis and Perry, 1994; Johnston, 2004), with most hens exhibiting a single characteristically long (prime) sequence about the time of peak egg production, which then declines at different rates between individuals (Robinson et al., 1990).

To reproduce these changes in sequence length over time, the internal cycle length initially needs to be long (usually more than 24 h), before decreasing with advancing time from first egg to close to, or below 24 h, and subsequently increasing. Internal cycle lengths longer than the external cycle length will cause the time of lay to be later each day, whereas those shorter than the external cycle length will enable the hen to lay long sequences with oviposition occurring at a similar time each day (Morris, 1978). Internal cycle lengths are under genetic control and can be manipulated (Foster, 1981); thus, the constraining effect of the external cycle length on potential rate of lay may be reduced either by reducing the internal cycle length or making use of ahemeral cycles greater in length than 24 h (Morris, 1973, 1978). External cycle lengths longer or shorter than 24 h can be accommodated when such an approach is used. When the ovulation curves of individuals in the flock are integrated, the characteristic laying curve is faithfully reproduced. The slope of the initial rise in flock egg production to peak rate of lay is influenced by the distribution of ages at sexual maturity and by the lengths of the individual prime sequences. The incidence of internal laying at onset of maturity plays a role in modifying rate of lay, but not ovulation rate. The persistency of lay after peak will be determined by the rate at which sequence lengths of individual hens shorten over time, as well as by the number of pause days. Hence, the prediction of sequence length is a logical step in predicting the performance of a flock of laying hens over an entire laying cycle.

In the model developed in Brazil, the equation adapted from Johnston and Gous (2006) and Ferreira et al. (2016) is used to describe changes in the internal cycle length after the first egg is laid. The difference between successive ovipositions, minus 24 h, is known as the lag between ovipositions. As each successive egg is laid, these lags will accumulate until a maximum of about 8 to 9 h, after which the hen will cease laying for a (pause) day and begin again the day after the pause. The total number of eggs laid between pauses is known as a clutch. The time of laying of the first egg of a sequence is controlled by the time that the photoperiod ends on the previous day, with a standard deviation of about 3 h. Subsequent eggs in a sequence are laid progressively later, and the time of laying of the last egg of the sequence is dictated by the cumulative lag (±0.2 h).

The reproductive rates of flocks of commercial laying hens and broiler breeders may be simulated by making use of the Monte Carlo simulation method, which requires the choice of appropriate values for the means and standard errors of the parameters in the various equations used to simulate ovulation rate and the rate of decay in internal cycle length. The incidence of internal laying and soft shelled eggs may also be accommodated (Johnston and Gous, 2006, 2007a, 2007b, 2007c). The potential performance of each hen in the population is simulated in this way, thereby producing information necessary for predicting the nutrients required by each hen on each day of lay. For more precision in determining these nutrient requirements, the weight of the egg and the proportions of yolk and albumen in the egg need to be known, and these can be modeled as described below.

Egg weight is estimated as the sum of its three components: yolk, albumen, and shell. Yolk weight has been shown to be a function of the age of the bird (McMillan et al., 1970; Nonis and Gous, 2013), which may differ between strains of laying hens. The parameters of the equations used to predict the weights of the three components were determined at UNESP (Ferreira et al., 2016; Bendezu et al., 2018).

According to Emmans and Fisher (1986) to predict the requirement of energy and amino acids to produce yolk, albumen, and shell, the content of each nutrient in each component is multiplied by the weight of each component: 2.5 kJ and 0.21 g of ideal protein per g of yolk, 2.6 kJ and 0.13 g of ideal protein per g of albumen, and 1.2 kJ and 0.004 g of ideal protein per g of shell. The requirement for each amino acid is calculated from the proportion of nitrogen in yolk (27 mg nitrogen/g) and albumen (17 mg nitrogen/g) (Lunven et al., 1973). As yolk deposition is a continuous process, it is assumed that 2 g of yolk is deposited each day. The efficiency of amino acid utilization from feed to egg protein was assumed to be 0.80 (Nonis and Gous, 2006).

## Predicting Maintenance Requirements

A large proportion of the daily intake of energy and amino acids by a laying hen or a broiler breeder hen is used for maintenance, so the prediction of the bird’s maintenance requirement, when determining her optimum daily intake of energy and amino acids, is of considerable importance. In most factorial models, these maintenance requirements are based on body weight, but because body lipid does not need to be maintained (Emmans and Fisher, 1986), a more accurate basis for calculating these requirements would be the body protein content of the bird.

In laying hens, it is well established that body protein content is maximal at sexual maturity and that little further protein growth occurs during lay (Fisher and Gous, 2008). It could be argued that broiler breeder hens are further from their somatically mature protein weight at sexual maturity than are laying hens, and hence that body protein growth may continue when the opportunity arises. Such might be the case in poor egg producers, where body protein may be deposited if the number of pause days becomes excessive, but there is no evidence to substantiate this. Provision for slow body weight gain in broiler breeder hens is often recommended, assuming a mean gain of about 650 g from 50% egg production to the end of lay.

However, as the weight of body protein remains relatively stable throughout the laying period, and as any growth in body protein may be regarded as taking place among nonlaying hens only, it should not be necessary to assume that protein growth is obligatory when determining nutrient requirements of laying hens or broiler breeder hens. Also, because changes in body lipid content are the consequence of the way in which the hen has been fed, it is unnecessary to make provision for any obligatory gain in body protein or lipid during lay. Maintenance requirements may thus be considered to be constant over the laying period for those birds that continue to lay in closed cycles, and these should be based on the body protein content of the bird at the age of first egg.

Where the simulation is started before all the hens have reached sexual maturity, the requirements for the growth of body protein, the ovary, and the oviduct need to be calculated as described by Bowmaker and Gous (1989) and Silva et al. (2014). Once each pullet starts laying, no further nutrients are assigned for growth. The total amount of amino acid and energy required per bird is the sum of the amounts required for yolk, albumen, shell, growth, and maintenance.

## Applying the Model to Determine Optimum Dietary Nutrient Contents

**Figure 2.**
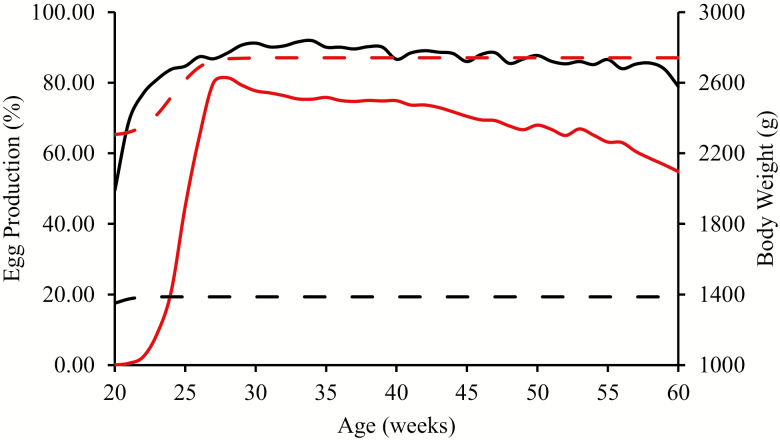
Simulated egg production (——) and body weight (– – –) of laying hens (black-lines) and broiler breeders (red lines) during the egg production period.

An egg production model was developed in MSExcel that simulates growth up to point-of-lay and daily egg production (rate of laying and egg weight) throughout a given laying cycle, from which the daily energy and amino acid requirements of a given laying hen and broiler breeder are calculated. Figure 2 and Table 1 show the results of such a simulation. It should be stressed that this is one example of a simulated output from the model and that virtually any laying curve and egg weight curve can be simulated using appropriate parameters.

**Table 1.** Mean simulated body protein weights and egg production of laying hens and broiler breeders within four periods of lay (20 to 60 wk)

			Production parameters		
Age (wk)	Strain	Body protein weight (g)	Rate of laying (eggs/100 birds)	Egg weight (g)	Egg output (g/bird d)
21 to 30	Layers	252	84	57.1	48
	Breeders	461	46	58.6	27
31 to 40	Layers	254	90	61.1	55
	Breeders	494	75	66.7	50
41 to 50	Layers	254	88	63.6	56
	Breeders	494	70	70.0	49
51 to 60	Layers	254	85	64.7	55
	Breeders	494	62	72.6	45

The maintenance requirements for broiler breeders will increase initially until all hens in the flock reach sexual maturity at about 30 wk, and these requirements will be higher than for laying hens because of their larger weight of body protein (Table 1). The feed intake of broiler breeders is restricted to an amount decided upon by the farm manager, so body weight, as opposed to body protein weight, may increase or decrease over time, depending on the amount of food allocated to the bird each day, as a result of changes in the amount of body lipid being stored or utilized.

**Figure 3.**
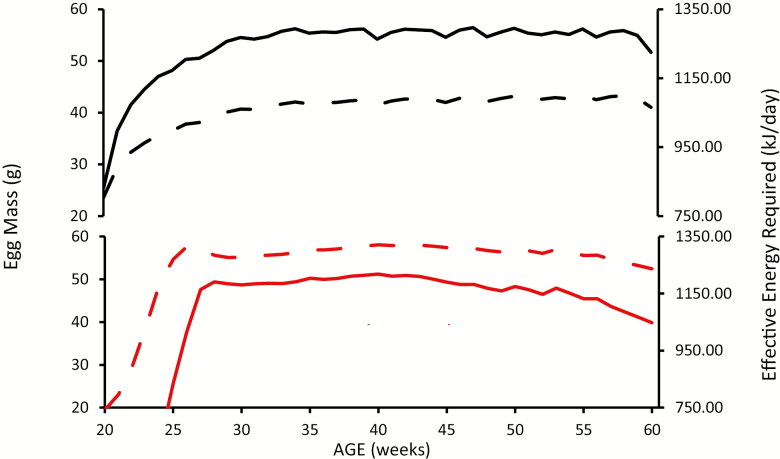
Simulated egg mass output (rate of lay × egg weight) (g/bird d) (——) and effective energy required (kJ/bird d) (– – –) over the laying period for laying hens (black lines) and broiler breeder (red lines).

It is evident that the laying patterns of commercial laying hens and broiler breeders differ (Figure 2), with laying hens having the potential to produce a far greater number of eggs than broiler breeders because of the intense selection that has been applied to improve rate of lay in commercial hens. This manifests in a higher peak rate of lay and a greater persistency. Egg weight increases over time but the rate of increase is greater in the case of broiler breeders (Table 1) contributing to the increased and differing requirement for nutrients with age. The simulated changes in egg output (g/bird d) and energy requirement for these two strains over the laying period are illustrated in Figure 3.

Determining the amino acid and energy requirements of a laying hen or broiler breeder by simulating the potential performance of these birds is the first step in deciding how to feed a flock of hens. Generating a flock of laying hens or broiler breeders and calculating the mean performance is likely to produce the same daily requirement for nutrients as for the average individual in the flock. The flock response provides information about the spread of laying performance and may be used to determine the optimum economic level of each of the essential nutrients in the feed; however, this requires prediction of the amount of food each hen will consume when presented with a given feed.

Where food intake is an input to the model, as is most often the case, it is naive to believe that feeding programs can be successfully optimized, because the composition of the food offered has important effects on voluntary food intake. Food intake must therefore be an output from, and not an input to, a model. The theory of food intake and growth proposed by Emmans (1981a, 1981b, 1989, 1997), and used in these models, is based on the premise that birds attempt to grow at their genetic potential, which implies that they attempt to eat as much of a given feed as would be necessary to grow and reproduce at that rate. The same principle can be applied to laying hens (Emmans and Fisher, 1986). By comparing the nutrient requirements, as calculated above, with the content of those nutrients in the feed, the “desired” feed intake can be determined: this is the amount of feed that would be needed to meet the requirement for the first limiting nutrient in the feed (Emmans, 1981b). The bird may not be capable of consuming this amount of feed because its intake may be constrained by bulkiness of the feed, the inability to lose sufficient heat generated to the environment, or the inability to deposit sufficient energy as body lipid due to a low potential fatness of the genotype. In this case, feed intake will be less than desired, and performance would be compromised.

This theory has been shown to predict food intake and hence growth and carcass composition with considerable accuracy (Ferguson and Gous, 1997, 2002; Ferguson et al., 1997; Wellock et al., 2004). Gous et al. (1987) and Burnham et al. (1992) have shown that broilers and laying hens increase food intake as the limiting nutrient in the feed is reduced, attempting thereby to obtain more of the limiting nutrient, until a dietary concentration is reached where performance is so constrained that food intake falls. The common misconception that “birds eat to satisfy their energy requirements” is too simplistic and of limited value in predicting voluntary food intake.

The critical features of a model to predict food intake in hens would be predictions of the body protein weight of the bird and its potential egg output on each day, from which nutrient requirements for maintenance and output may be calculated; a description of the nutrient content of the feed on offer; and a description of the effective temperature of the environment in which the bird is housed. Although the principle of predicting food intake is the same for growing and reproducing birds, the description of potential growth and of egg output differs markedly between the two, as is evident from the above.

The situation with broiler breeder hens differs from that of full-fed laying hens in that a daily allowance of feed is allocated, this being less than would normally be consumed if the birds were given ad libitum access to feed. Yet the principles applied to the prediction of voluntary food intake, described above, remain. The difference is that the desired food intake of the birds may not always be achieved; thus, the actual food intake would be that constrained by the farm manager. Consequently, egg output will be a function of the amount of limiting nutrient remaining after the maintenance requirement of the hen has been met. Whether the consequences of underfeeding are more likely to be evident with broiler breeders than with commercial laying hens, given that laying hens are fed ad libitum, would depend on the daily amount of food allocated to the breeders in relation to their potential egg output, the density of the feed allocated to the laying hens, and the environmental temperature to which they are subjected.

The response of an individual hen to increasing levels of a limiting nutrient is usually linear to the point where the genetic potential is reached (Fisher et al., 1973) with no further increase in output at higher intakes. Laying hen nutritionists are interested in responses to nutrients in economically important outputs such as egg output, number of chicks produced per hen, and so on. Because such responses are usually measured using groups of birds, they are invariably curvilinear, being the result of integrating the responses of individuals making up that population (Fisher et al., 1973). Populations of birds therefore cannot have “requirements” for nutrients. Nutritionists seek the optimum economic dietary contents of each nutrient, and for this, they need to know how populations respond to increasing dietary contents of essential nutrients. Descriptions of such responses, while taking account of marginal costs and revenues, are therefore invaluable in determining how to maximize or minimize the objective function chosen for any given commercial operation. Simulation modeling can provide this information.

**Figure 4.**
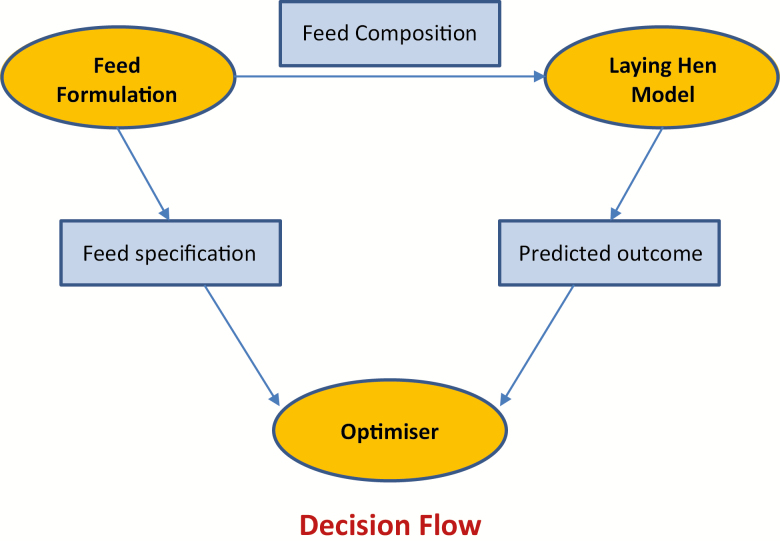
Decision flow in determining the optimum economic nutrient levels to include in a laying hen or broiler breeder feed.

The process of optimization is illustrated in Figure 4. A feed is formulated and passed to the laying hen model where performance is simulated and costs and revenues are calculated. The optimizer then alters the feed formulation using certain rules, and the process is repeated until no further improvement can be made to the value of the objective function. By implementing the results of such an optimization process, egg producers have a greater chance of maximizing profitability for their enterprise than by relying on tables of nutrient requirements and least cost feed formulation.

## Conclusions

In the models of commercial laying hens and broiler breeders described here, the theory is applied to each individual making up the population using appropriate means and standard errors for specific parameters to produce a stochastic model. The responses are acceptable representations of reality and are ideal for determining the optimum economic method of feeding these simulated flocks. A simulation modeling approach can eliminate the need for expensive, long-term laying trials designed to measure the response of laying hens or broiler breeders to energy and protein.

## About the Authors

**Dr. Sakomura** is a full professor in the Department of Animal Science in the College of Agriculture and Veterinary Sciences at Sao Paulo State University (UNESP). She earned a PhD in poultry nutrition at the Universidade Federal de Viçosa and completed postdoctoral research in poultry nutrition at the University of Arkansas (USA). Currently, she is animal science coordinator at the São Paulo Research Foundation (FAPESP) in Brazil. Dr. Sakomura has dedicated her main research to nutritional modeling in poultry, focusing on energy and amino acid requirements, including mathematical models to predict growth and egg production. She has also developed software to simulate growth, egg production, and nutrient requirements for poultry.

**Dr. Reis** graduated from the Universidade Federal de Lavras in 2016 after spending 2014 at the University of Delaware (USA) where he evaluated the effect of probiotics on gene expression in broiler chickens. He is currently a postdoctoral fellow in mathematical modeling applied to animal nutrition at the Department of Animal Science in the College of Agriculture and Veterinary Sciences at Sao Paulo State University (UNESP). His main research interests are monogastric nutrition and modeling as a tool to describe biologic phenomena to optimize poultry systems.

**Dr. Ferreira** graduated from Sao Paulo State University (UNESP), where she studied growth and egg production in broiler breeders. She was a postdoctoral fellow in the Department of Animal Science in the College of Agriculture and Veterinary Sciences at Sao Paulo State University (UNESP) and at the University of KwaZulu-Natal, South Africa, where she developed a stochastic model to predict egg production and requirements of laying hens and broiler breeders. Her research interests are in poultry nutrition and mathematical modeling. Currently, Dr. Ferreira is a poultry regional manager at Polinutri Alimentos S.A. in Brazil.

After an academic career spanning 37 yr, **Dr. Rob Gous** retired from the University of KwaZulu-Natal, South Africa, in 2007 and is now an emeritus professor and senior research fellow. His research interests are in the development of simulation models for growing broilers, turkeys, and pigs and for laying hens and broiler breeders.
